# Sleep quality and its impacts on quality of life among military personnel in remote frontier areas and extreme cold environments

**DOI:** 10.1186/s12955-020-01460-7

**Published:** 2020-07-13

**Authors:** Zonghua Wang, Beijing Chen, Wei Li, Fei Xie, Alice Yuen Loke, Qin Shu

**Affiliations:** 1 School of Nursing, Army Medical University, Chongqing, 400038 China; 2 School of Nursing, the Hong Kong Polytechnic University, Hung Hum, Kowloon, Hong Kong, China

**Keywords:** Sleep, Military, PSQI, HRQoL

## Abstract

**Background:**

Poor sleep quality negatively affects the readiness of military operations and is also associated with the development of mental health disorders and decreased quality of life. The purpose of this study was to investigate the sleep quality of military personnel from remote boundaries of China and its relationship with coping strategies, anxiety, and health-related quality of life (HRQoL).

**Methods:**

A cross-sectional survey was performed among military officers and soldiers from a frontier defence department and an extreme cold environment. The participants were surveyed using the Pittsburgh Sleep Quality Index (PSQI), Trait Coping Style Questionnaire (TCSQ), Self-rating Anxiety Scale (SAS), and Short Form Health Survey (SF-36).

**Results:**

A total of 489 military officers and soldiers were included. The participants had a mean age of 22.29 years. The median overall PSQI score was 7.0 (IQR, 4.0 ~ 9.0), with 40.9% (200/489) of the subjects reporting poor sleep quality. The difficulties with sleep were mainly related to daytime dysfunction due to disrupted sleep, sleep latency, and subjective sleep quality. The median score of the SF-36 physical component was 83.5 (IQR, 73.0 ~ 90.5), and the median score of the mental component was 74.1 (IQR, 60.4 ~ 85.1). Significant correlations were found between the PSQI and SF-36 (*r* = − 0.435, *P* <  0.01). Anxiety symptoms, marital status, educational background, and global PSQI score were demonstrated as predictors of a low SF-36 physical component by multiple regression analysis (*F* = 17.06, *P* <  0.001, R^2^ = 0.117).

**Conclusions:**

Sleep difficulty is a prevalent and underestimated problem in the military that negatively influences HRQoL, especially in physical and social functioning. Evaluation of and education on pain were recommended because of body pain and its negative impacts on sleep quality, coping strategies, anxious emotions and HRQoL.

## Background

Sleep loss and sleep disruption are prevalent among military service members. Military operations are stressful, unexpected, urgent, and often take place in austere environments, predisposing soldiers to insufficient and poor quality sleep. A study reported a dramatic increase in clinically significant sleep disorders among officers who were involved in the combat operation of Operation Iraqi Freedom [1]. A study in the United States reported that nearly half of military personnel were screened as positive for disturbed sleep. However, inadequate and poor quality sleep is regarded as ‘normal’ and ‘unavoidable’ among military personnel because of the nature of military operations and special missions that frequently require shift work, long-term field training, and rapid deployment across multiple time zones [2]. Sleep disorders and their negative impacts are commonly underestimated because in the military, missions are always the priority, and there is no chance to consider sleep.

The promotion of healthy sleep is critical to the physical and behavioural health of individual military members as well as to the functioning of the entire unit [3]. Emerging evidence suggests that sleep difficulties and/or sleep disruptions have been associated with the development of mental health disorders, including depression, anxiety, post-traumatic stress disorder (PTSD), and eventually, a decrease in health-related quality of life (HRQoL) [4–9]. In particular, poor sleep quality can negatively affect military operational readiness, safety and effectiveness through reduced cognitive performance, such as impaired attention, concentration, responsiveness, judgement, and critical thinking [10, 11].

However, very few studies have been conducted on sleep quality and its impacts on HRQoL among the Chinese military population, particularly among those who served at the country borders and in extreme cold environments [12, 13]. Military personnel who serve at borders, which are challenged by both hostile environments in remote areas with disputes over border and religious conflicts, are vulnerable to poor sleep quality and HRQoL. This study attempted (1) to investigate the current status of sleep quality among military service members at remote boundaries and in extreme cold environments in China; (2) to explore the association between poor sleep quality and HRQoL; and (3) to identify factors that could affect sleep quality and HRQoL.

## Methods

### Study design and participants

This was a cross-sectional study. Data on sleep quality, coping strategies, anxiety level, and HRQoL were collected with self-administered questionnaires. Five hundred military officers and soldiers were recruited from a frontier defence department in February 2018. The frontier defence department is located in a city in northwestern China. The city name literally means ‘new frontier’ or ‘new territory’ in Chinese language, and the city occupies roughly one-sixth of China’s total area. Featuring vast, inhospitable desert and massive snow-capped mountains, the city is known by geographers as ‘the dead heart of Asia’ and China’s ‘Wild West’. It has a 5600 km boundary and is challenged by border disputes and religious conflicts. The temperature during winter was 20 degrees below zero on average. Military personnel were considered eligible for inclusion if they were (1) aged 18 to 60 years, (2) able to read, write and speak Mandarin, (3) willing to participate in the study, and (4) able to give informed consent.

### Questionnaire

The questionnaire consisted of five parts: demographics, the Pittsburgh Sleep Quality Index [14], the Self-rating Anxiety Scale [15], the Trait Coping Style Questionnaire-Chinese version [16], and the Chinese version of the Medical Outcomes Study Short Form-36 [17].

Part 1 was to solicit demographic information about the participants, including age, sex, marital status, education level, and years of service in the military.

Part 2 consisted of the Pittsburgh Sleep Quality Index (PSQI) [14], which was used to assess participant sleep quality and disturbances over a one-month period. The PSQI is composed of 19 items in seven components: subjective sleep quality (SQ), sleep latency (SL), sleep duration (SD), habitual sleep efficiency (SE), sleep disturbances (SB), the use of sleep medication (SM), and daytime dysfunction due to disrupted sleep (DD) [18]. The items were rated on a 3-point Likert scale with ‘0’ indicating no difficulty and ‘3’ indicating severe difficulty. For each item, scores ≥ 2 indicated poor sleep quality. The overall PSQI score ranges from 0 to 21, with higher scores indicating poorer sleep quality and more sleep disturbances. The Cronbach’s α coefficient of the Chinese PSQI was 0.71 ~ 0.83 [19, 20], indicating acceptable levels of reliability. In our participants, the Cronbach’s α value was 0.78. The PSQI is a self-reported questionnaire that distinguishes ‘a good sleeper’ as having a PSQI global score ≤ 7 and ‘a poor sleeper’ as having a PSQI score ≥ 8 [21].

Part 3 was the Self-rating Anxiety Scale (SAS) [15], which was used to explore military officers’ anxiety-related complaints. The SAS is a 20-item 4-point Likert scale assessing both affective and somatic symptoms, with 1 = a little of the time and 4 = most of the time. The raw scores, which range from 20 to 80, are converted to standard scores by dividing the sum of the raw scores by 80 and multiplying by 100 [22, 23]. The higher the overall score, the more frequently anxiety symptoms are experienced. The internal consistency coefficient of the Chinese version of the SAS was 0.84 [24]. In our subjects, the Cronbach’s α score was 0.77. SAS standard scores greater than 50 indicate clinically significant anxiety [25, 26].

Part 4 was the Trait Coping Style Questionnaire-Chinese version (TCSQ) [16], which was used to assess the coping strategies of the participants. The TCSQ was developed based on a review of the existing literature and a content analysis of coping strategies and adopted for use in the Chinese population [27]. The scale consists of 20 items rated on a 5-point Likert scale and evaluates two components: positive coping style (10 items) and negative coping style (10 items). The Cronbach’s α coefficients of the positive and negative coping subscales were 0.79 and 0.78, respectively [28]. In our sample, the internal consistency values of the positive and negative subscales were 0.72 and 0.80, respectively.

Part 5 was the Chinese version of the Medical Outcomes Study Short Form-36 (MOS-SF-36) [17], which was used to assess HRQoL. The MOS-SF-36 includes 36 items that correspond to eight domains grouped into two components: the physical component summary (PCS) includes general health (GH); role limitations due to physical health (role-physical, RP); bodily pain (BP); and physical functioning (PF). The mental component summary (MCS) includes mental health (MH, also known as emotional well-being); vitality (VT, also known as energy/fatigue); role limitations due to emotional problems (role-emotion, RE); and social functioning (SF). Each item of the SF-36 is assessed using points scored on a Likert scale [29]. The raw scores of each of the eight domains are calculated by summing the scores of all items and then a transformed score is calculated as follows: transformed score = (actual raw score - lowest possible raw score)/(possible max raw score) * 100. The PCS and MCS scores range from 0 to 100 points, with higher scores indicative of better HRQoL. The Cronbach’s α score of each domain ranges from 0.39 to 0.88 [30]. In our participants, the Cronbach’s α score of each domain ranged from 0.51 to 0.89, with a Cronbach’s α coefficient for the overall scale of 0.82.

### Data collection

The data were collected after a seminar, and the questionnaires were administered anonymously. This study was conducted in accordance with the ethical principles of the Declaration of Helsinki.

### Ethical considerations

Ethical approval was obtained from the human ethics committee of the Army Medical University.

### Data analysis

Statistical Package for the Social Sciences 21.0 (SPSS Inc., Chicago, IL) were adopted to perform the data analysis. The descriptive statistics (median, interquartile range [IQR], number and percentage) were calculated. A normal distribution was not observed in our variables with the Kolmogorov-Smirnov test. The Mann-Whitney U test or Kruskal-Wallis test was used to compare two or more variables for the PSQI and HRQoL.

Correlations between variables were investigated with Spearman’s correlation coefficient. According to Ratner [31], a coefficient below 0.3 represents a weak correlation, a coefficient between 0.3 and 0.7 means a moderate correlation, and a coefficient higher than 0.7 corresponds to a high correlation.

Multivariate linear regression analysis (stepwise regression) was performed to determine the impacts of sleep quality on HRQoL. A *p*-value less than 0.05 was considered statistically significant.

## Results

### Sample characteristics

Five hundred military officers and soldiers were randomly recruited from a military unit in February 2018. A total of 498 questionnaires were returned, and nine of them were excluded because of 50% or more items missing data. As a result, only 489 questionnaires were included in the final analysis.

The 489 participants were 22.3 years old (ranging from 18 to 38) on average. The majority of them were males (*n* = 478, 97.8%) and enlisted military personnel (*n* = 417, 85.3%). Among them, 11 (2.2%) were health care workers, 38 (7.8%) were administrative workers, and 440 (90.0%) were soldiers who received training in snow hunting in the Gobi Desert and in counterterrorism combat in a depopulated zone.

The participants had served in the military for an average of 35.3 months, ranging from 4 to 217 months. Approximately two-thirds (*n* = 317) had served for less than 3 years, one-fifth (*n* = 99) had served for 3 to 6 years, and only 41 of them (9%) had served for 6 to 9 years.

### Anxiety

The anxiety scores of our study participants ranged from the lowest score of 25.0 to the highest score of 81.3, with a median score of 40.0 (IQR, 35.6 ~ 45.0). The participants with a habit of drinking showed a higher level of anxiety than those without a habit of drinking (*P* = 0.046) (Table 1). The participants with a high level of anxiety (SAS score >  50) had worse HRQoL in terms of bodily pain (*P* <  0.01) and physical functioning (*P* = 0.010) (Table 3).

**Table 1:** Comparisons on the scores of PSQI, anxiety and coping styles by different individual characteristics

Variables	n (%)	Anxiety	global PSQI	SQ	SD	SE	SB	DD	PC	NC
		median(IQR)								
**Age (years)**										
≤ 20	168(34.3)	40(36.3–46.3)	6.9(4–9.8)	1(1–2)	1(0–1)	0(0–1)	1(0.9–1)	2(1–3)	35(31–39)	26(18.3–31)
21 ~ 30	306(62.6)	40(35–43.8)	7(4–9)	1(1–2)	1(0–1)	0(0–1)	1(1–2)	2(1–3)	35(31–39)	25(19–30)
> 30	15(3.1)	40(35–48.8)	7(5–10)	1(1–2)	1(1–2)	1(0–1)	1(1–2)	2(1–2)	34(30.8–36.3)	26(23–31)
*P* value		0.544	0.832	0.966	0.002	0.047^*^	0.713	0.611	0.282	0.819
**Gender**										
Male	478(97.8)	40(36.3–45)	7(4–9)	1(1–2)	1(0–1)	0(0–1)	1(1–1)	2(1–3)	35(31–39)	25(19–30)
Female	11(2.2)	40(32.5–48.8)	8(6–10)	2(1–2)	1(1–1)	0(0–0)	1(1–2)	2(2–3)	37(33–39)	28(22–31)
*P* value		0.844	0.294	0.386	0.391	0.329	0.093	0.226	0.394	0.355
**Marriage**										
Unmarried	459(93.9)	40(35–45)	7(4–9)	1(1–2)	0(0–1)	0(0–1)	1(1–1)	2(1–3)	35(31–39)	25(19–30)
Married	30(6.1)	38.8(35.9–49.1)	7(5–9)	1(1–2)	1(1–2)	0(0–1)	1(1–1)	2(1–3)	34(30–37)	26.5(19.8–31.3)
*P* value		0.909	0.559	0.992	0.005^**^	0.468	0.239	0.320	0.202	0.414
**Education**										
Primary	24(4.9)	42.5(40–54.4)	6.5(4.3–10)	1(0.3–2)	1(1–1.8)	0(0–1)	1(1–1)	2(1–2.8)	33(30–35)	26(20.3–31.5)
Middle	243(49.7)	40(35–45)	6(3–9)	1(1–2)	1(0–1)	0(0–1)	1(0–1)	1(0–2)	35(31–39)	25(18–30)
College/University	147(30.1)	40(36.3–46.3)	7(4–10)	1(1–2)	1(0–1)	0(0–1)	1(1–1)	2(1–3)	35(31–39)	26(19–31)
Postgraduates	75(15.3)	38.8(35–43.8)	7(5–8)	1(1–2)	1(1–2)	0(0–1)	1(1–1)	2(1–3)	36(32–40)	26 (19–30)
*P* value		0.107	0.055	0.244	0.000^**^	0.997	0.000^**^	0.009^**^	0.210	0.552
**Current Drinker**										
Yes	36(54.0)	41.9(38.8–54.4)	8.5(6–12)	1(1–2)	1(1–1)	0(0–1)	1(1–1)	2(1–3)	32.5(27.8–39)	26(23.3–32.8)
No	453(46.0)	40(35–45)	7(4–9)	1(1–2)	1(0–1)	0(0–1)	1(1–1)	2(1–3)	35(32–39)	25(18–30)
*P* value		0.004^**^	0.003^**^	0.053	0.251	0.441	0.006^**^	0.081	0.046^*^	0.031^*^
**Current Smoker**										
Yes	264(54.0)	40(36.3–45)	7(5–9)	1(1–2)	1(0.3–1)	0(0–1)	1(1–1)	2(1–3)	35(31.3–39)	26(19–31)
No	225(46.0)	40(35–45)	6(3.5–9)	1(1–2)	1(0–1)	0(0–0.2)	1(0.9–1)	2(1–3)	35(31–39)	25(18–29)
*P* value		0.235	0.071	0.023^*^	0.065	0.012^*^	0.951	0.900	0.941	0.047^*^

*PSQI* Pittsburgh sleep quality index, *SQ* subjective sleep quality, *SD* sleep duration, *SE* habitual sleep efficiency, *SB* sleep disturbances, *DD* daytime dysfunction due to disrupted sleep, *NC* negative coping, *PC* positive coping

n = Frequency; % = Percentage, *IQR* Interquartile range; ^**^P <  0.01; ^*^P <  0.05

### Positive and negative coping style

The median score for negative coping style was 25.0, with an IQR from 19.0 to 30.0. The median score for positive coping style was 35.0, with an IQR from 31.0 to 39.0. Drinking and smoking were significantly associated with negative coping styles. Participants who drank had a higher level of negative coping style than non-drinkers (*P* = 0.031). Participant who smoked had a higher level of negative coping style than non-smokers (*P* = 0.047) (Table 1).

### Sleep quality

The median score for the overall PSQI was 7.0 (IQR, 4.0 ~ 9.0), with 40.9% (200/489) of the subjects reporting poor sleep quality. The percent of participants who had domain scores ≥2 were as follows: daytime dysfunction due to disrupted sleep, 56.1% (274/489); sleep latency, 45.8% (224/489); subjective sleep quality, 35.6% (174/489); sleep duration, 13.5% (66/489); sleep disturbances, 11.9% (58/489); habitual sleep efficiency, 6.1% (30/489); and the use of sleep medication, 0.4% (2/489).

Compared to a sample of Chinese inland army members (*n* = 314) [32], the study participants had significantly higher scores in the SQ (1.28 vs 0.86, *P* <  0.01), SL (1.71 vs 1.22, *P* <  0.01), and DD (1.63 vs 0.78, *P* <  0.01) domains and in the total PSQI score (6.76 vs 5.69, *P* <  0.01) (Table 2).

**Table 2:** Comparisons of SF-36 and PSQI scores between participants and Chinese norms

Variables		Study group (*n* = 489)	Chinese norms^a^ (aged 18–44 years) (*n* = 876)	Chinese Armed force^b^ (aged 17–36 years) (*n* = 314)	*P* value
**SF-36**	GH	77.2 ± 20.8	60.0 ± 19.8		< 0.01
	RP	82.7 ± 32.8	85.3 ± 29. 0		0.13
	BP	70.5 ± 19.6	85.0 ± 17.8		< 0.01
	PF	92.6 ± 14.7	86.0 ± 18.0		< 0.01
	MH	67.8 ± 17.6	57.9 ± 21.4		< 0.01
	VT	64.7 ± 19.1	53.3 ± 20.3		< 0.01
	RE	72.8 ± 39.7	85.3 ± 30.5		< 0.01
	SF	78.4 ± 19.4	84.2 ± 16.9		< 0.01
**PSQI**	SQ	1.28 ± 0.95		0.86 ± 0.79	< 0.01
	SL	1.71 ± 1.68		1.22 ± 0.95	< 0.01
	SD	0.87 ± 0.67		0.86 ± 0.92	0.86
	SE	0.38 ± 0.63		0.93 ± 1.11	< 0.01
	SB	0.89 ± 0.60		0.97 ± 0.59	0.06
	SM	0.01 ± 0.14		0.06 ± 0.26	< 0.01
	DD	1.63 ± 1.08		0.78 ± 0.89	< 0.01
	Total	6.76 ± 3.55		5.69 ± 3.31	< 0.01

*SF-36* 36-item Short form health survey, *PSQI* Pittsburgh sleep quality index, *GH* general health, *RP* role-physical, *BP* bodily pain, *PF* physical functioning, *MH* mental health, *VT* vitality, *RE* role-emotional, *SF* social functioning, *SQ* subjective sleep quality, *SL* sleep latency, *SD* sleep duration, *SE* habitual sleep efficiency, *SB* sleep disturbances, *SM* the use of sleep medication, *DD* daytime dysfunction due to disrupted sleep

^a^normative data from a sample of citizens in Hangzhou of China

^b^normative data from a sample of Chinese inland army

### Quality of life

The median score for the SF-36 physical component was 83.5 (IQR, 73.0 ~ 90.5), and the median score for the SF-36 mental component was 74.1 (IQR, 60.4 ~ 85.1). Table 2 presents the comparisons of the HRQoL scores of our participants with the normative data. The participants of this study had statistically higher scores in the following four domains: GH (77.2 vs 60.0, *P* <  0.01), PF (92.6 vs 86.0, *P* <  0.01), MH (67.8 vs 57.9, *P* <  0.01) and VT (64.7 vs 53.3, *P* <  0.01) and showed significantly lower scores in the following three domains: BP (70.5 vs 85.0, *P* <  0.01), RE (72.8 vs 85.3, *P* <  0.01) and SF (78.4 vs 84.2, *P* <  0.01) (Table 2). The normative data were from a Chinese population in Hangzhou city and obtained by cluster sampling [33]. A total of 1688 responses were received and divided into three subgroups: a young and middle-aged group under the age of 45 years old, a middle-aged group between the age of 45 and 64 years old, and an adult group older than 65 years.

### Differences in sleep quality and HRQoL according to different individual characteristics

As shown in Table 1, significant differences were found in the scores for global PSQI (*P* = 0.003) and SB (*P* = 0.006) between participants who had a habit of drinking and those who did not. Participant who smoked had a higher level of sleep disorders, including SQ (*P* = 0.023) and SE (*P* = 0.012). Participants with higher education levels had a significantly higher level of sleep disorders, including SD (*P* <  0.01), SB (*P* <  0.01) and DD (*P* <  0.01).

Table 3 displays the comparisons of median scores for the SF-36 domains according to different individual characteristics. The married participants had worse HRQoL regarding physical functioning (*P* = 0.008) and role limitations due to physical health (*P* = 0.014) than unmarried participants. The participants who had a habit of drinking had worse HRQoL in terms of bodily pain than those who did not drink (*P* = 0.032). The participants who were smokers had worse HRQoL in terms of vitality (energy/fatigue) than those who were non-smokers (*P* <  0.05).

**Table 3:** Comparisons on SF-36 subscales among different variables

Variables	n (%)	GH	RP	BP	PF	MH	VT	RE	SF
		median(IQR)							
**Marriage**									
Unmarried	459(93.9)	82(65–97)	100(75–100)	72(62–84)	100(95–100)	68(56–80)	65(50–80)	100(33.3–100)	87.5(75–87.5)
Married	30(6.1)	68.5(60–93.3)	87.5(43.8–100)	62(59.3–74)	95(90–100)	70(48–81)	70(55–80)	100(25–100)	75(62.5–87.5)
*P* value		0.106	0.014*	0.060	0.008**	0.887	0.559	0.980	0.301
**Current Drinker**									
Yes	36(54.0)	81(65–97)	100(75–100)	62(51–73.5)	95(86.3–100)	68(52–84)	60(51.3–75)	100(66.7–100)	75(62.5–87.5)
No	453(46.0)	82(62–97)	100(75–100)	72(62–84)	100(95–100)	68(56–80)	65(50–80)	100(33.3–100)	87.5(75–87.5)
*P* value		0.902	0.809	0.032*	0.082	0.754	0.385	0.343	0.343
**Current Smoker**									
Yes	264(54.0)	82(65–97)	100(75–100)	72(62–84)	100(95–100)	68(56–80)	65(50–80)	100(33.3–100)	87.5(75–87.5)
No	225(46.0)	82(60–97)	100(75–100)	72(62–84)	100(94.3–100)	68(56–80)	70(55–80)	100(66.7–100)	87.5(75–87.5)
*P* value		0.939	0.998	0.912	0.684	0.449	0.016*	0.521	0.206
**Sleep quality**									
Good sleeper (≤ 7 PSQI)	289(59.1)	82(65–97)	100(75–100)	72(62–100)	100(95–100)	68(56–80)	70(55–80)	100(33.3–100)	87.5(75–87.5)
Poor sleeper (> 7 PSQI)	200(40.9)	82(60–92)	100(75–100)	62(51–72)	95(90–100)	68(56–80)	65(50–78.8)	100(41.7–100)	78.7(62.5–87.5)
*P* value		0.100	0.477	0.000**	0.000**	0.550	0.041*	0.746	0.114
**Anxiety**									
Without (≤ 50 SAS)	426(84.9)	82(62–97)	100(75–100)	72(62–84)	100(95–100)	68(56–80)	65(50–80)	100(33.3–100)	87.5(75–87.5)
With (> 50 SAS)	63(15.1)	77(60–97)	100(50–100)	62(50–72)	95(80–100)	68(52–84)	70(50–80)	100(66.7–100)	87.5(62.5–87.5)
*P* value		0.546	0.098	0.000**	0.010*	0.912	0.782	0.952	0.567

*SF-36* 36-item Short form health survey, *PSQI* Pittsburgh sleep quality index, *SAS* Self-rating anxiety scale, *GH* general health, *RP* role-physical, *BP* bodily pain, *PF* physical functioning, *MH* mental health, *VT* vitality, *RE* role-emotional, *SF* social functioning

n = Frequency; % = Percentage; ^**^P < 0.01; ^*^P < 0.05

### Comparisons of HRQoL among good sleepers and poor sleepers

Based on the PSQI global score, the sample was divided into two groups: good sleepers (scores ≤7) (*n* = 289) and poor sleepers (score ≥ 8) (*n* = 200). The good sleepers demonstrated better HRQoL in terms of bodily pain (*P* <  0.01), vitality (*P* = 0.041) and physical functioning (*P* <  0.01) (Table 3). A significantly higher physical component score (PCS) was found among good sleepers than among poor sleepers (*P* <  0.01), while no significant difference between the two groups was found regarding the mental component score (MCS) (*P* = 0.26).

### Associations between various variables

There were significant moderate correlations between the PSQI and bodily pain (BP) (*r* = − 0.435, *P* <  0.01), the PSQI and the SAS (*r* = 0.469, *P* <  0.01), the PSQI and NC (*r* = 0.382, *P* <  0.01), BP and the SAS (*r* = 0.423, *P* <  0.01), and BP and NC (*r* = 0.304, *P* <  0.01) (Table 4).

**Table 4:** Correlation coefficients between various variables

Variables	Role-physical	Mental health	Vitality	Role-emotional	Social functioning	Negative coping	Anxiety	Sleep quality
General health	0.301^**^	0.352^**^	0.328^**^	0.295^**^	0.402^**^	−0.105^**^	–	–
Role-physical	–	0.141^**^	0.208^**^	0.431^**^	0.344^**^	–	–	–
Bodily pain	–	–	–	–	0.117^**^	−0.304^**^	− 0.423^**^	− 0.435^**^
Mental health	–	–	–	–	0.470^**^	–	–	–
Vitality	–	0.636^**^	**–**	–	0.492^**^	–	–	–
Role-emotional	–	0.356^**^	0.328^**^	–	0.430^**^	–	–	–
Positive coping	–	–	–	–	–	−0.302^**^	−0.417^**^	–
Negative coping	–	–	–	–	–	–	0.546^**^	−0.382^**^
Anxiety	–	–	–	–	–	–	–	−0.469^**^

Only significant correlations were presented. ^**^P < 0.01; The correlation coefficient > 0.3 were presented in bold

### Sleep quality independently affects the PCS and the SAS

As shown by the multiple regression results in Table 5, the PCS was affected by anxiety symptoms, marital status, educational background, and global PSQI score (*F* = 17.06, *P* <  0.001, R^2^ = 0.117), and poor sleep quality was an independent predictor of low PCS. Anxiety was significantly affected by coping strategies, global PSQI score and education level, and poor sleep quality was also a significant predictor for developing anxiety symptoms (*F* = 70.49, *P* <  0.001, R^2^ = 0.364).

**Table 5:** Multiple linear regressions of predictors to HRQoL and Anxiety

Variables	B ± SE	Beta (β)	*t*	*P* value
**PCS (adjusted R**^**2**^ **= 0.117)**				
Constant	109.408 ± 3.89		28.129	< 0.001
Anxiety	−0.307 ± 0.67	− 0.213	−4.559	< 0.001
Marriage	−7.310 ± 2.36	−0.134	−3.093	0.002
PSQI	−0.505 ± 0.17	−0.137	−2.927	0.004
Education	−1.865 ± 0.71	−0.115	− 2.628	0.009
**Anxiety (adjusted R**^**2**^ **= 0.364)**				
Constant	42.581 ± 2.59		16.470	< 0.001
NC	0.421 ± 0.05	0.359	9.039	< 0.001
PC	−0.369 ± 0.05	− 0.256	−6.891	< 0.001
PSQI	0.579 ± 0.10	0.226	5.757	< 0.001
Education	−1.020 ± 0.41	−0.091	−2.502	0.013

*PSQI* Pittsburgh sleep quality index, *PCS* physical component summary, *NC* negative coping, *PC* positive coping

### Conceptual model of sleep quality and HRQoL

**Fig. 1:**
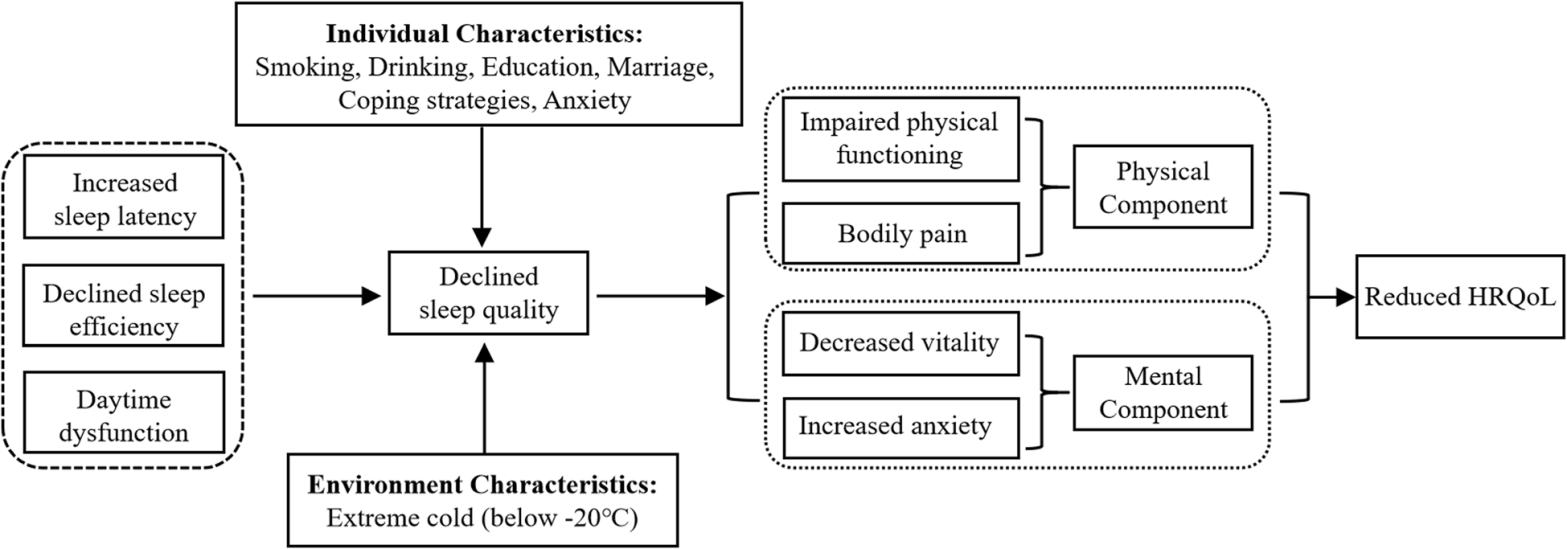
Conceptual model of impaired sleep quality and its impacts on decreased HRQoL

A conceptual model of how sleep quality impacts HRQoL among military personnel was developed according to the Wilson and Cleary model [34] for HRQoL. As shown in the conceptual model (Fig. 1), individual characteristics (e.g., smoking, drinking, education, marital status, coping strategies, anxiety) and environmental characteristics (extreme cold) are factors impairing soldiers’ sleep quality. The symptoms of impaired sleep quality included increased sleep latency, decreased sleep efficiency and daytime dysfunction due to insufficient sleep. These symptoms had a wide-ranging detrimental impact on physical and mental functioning, which consequently contributed to the decline in HRQoL.

## Discussion

The present study demonstrated poorer HRQoL in the military population regarding pain, role limitations and social functioning than in the normative data from the general population but better HRQoL in terms of general health, physical functioning, emotional well-being and vitality. Sleep quality and anxiety are contributing factors that influence physical-related quality of life. Moreover, the poor sleepers exhibited more impaired HRQoL in relation to physical complaints than the good sleepers, suggesting that sleep had a greater impact on the physical health component than on the mental health component. In terms of recommendations for improving HRQoL in military personnel, health interventions should be given not only to reduce emotional disorders such as anxiety but also to control physical symptoms including pain and sleep disorders and to improve social participation and communication.

The results may be challenged by the traditional beliefs that military personnel are at high risk of psychiatric problems because they are facing more stressful military tasks and a more austere environment. However, evidence is emerging that military troops tend to have better psychological resilience than civilians and volunteers. Military and police officers are relatively less likely to suffer from PTSD than civilians [35]. One reason for this phenomenon, we assumed, is that individuals who choose to take on military roles tend to have solid beliefs and spirits devoted to the nation and people, and they tend to feel great responsibility for national defence, so they present high resilience in the face of difficulties [36]. Another reason relies on the increasing awareness and implementation of programmes on stress management and resilience to military personnel due to their high risks of mental disorders. This training helps them be better prepared to handle psychological threats than civilians who seldom have the opportunities to be trained [37–39]. Moreover, military officers are frequently exposed to complicated situations during daily training so they are familiar with stress management strategies through repetitive practice.

This study contributed to previous findings by showing that sleep disorder was a prevalent problem among troops located in austere environments, especially in terms of poor subjective sleep quality, prolonged sleep latency and daytime impairment. One study among island garrisons revealed an overall PSQI score of 7.58 ± 2.96, with 45.7% of the subjects reporting poor sleep quality [40]. Sleep latency, daytime dysfunction, sleep disturbances, and sleep efficiency were reported as prominent problems among the island troops. Another study among Chinese inland armed forces disclosed an overall PSQI score of 5.69 ± 3.28, and one in four had poor sleep quality. The primary problems were sleep latency, sleep efficiency, vitality and daytime dysfunction. Kong and associates found a global PSQI score of 8.14 ± 3.79 among young soldiers stationed on the Tibetan Plateau [11]. Several reasons for sleep problems in austere environments include noise, extreme temperatures, frequent shift work, and crowded sleeping areas [41]. Moreover, servicemen undergoing multiple combat deployments were more likely to be exposed to the conditions that disrupted sleep than servicemen undergoing fewer deployments. A survey of service members and veterans of Operation Enduring Freedom and Operation Iraqi Freedom showed clinically significant poor sleep quality, with a mean global PSQI score of 10.36 ± 4.55. The mean score resembled normative data among poor sleepers or those with sleep disorders. Other than objective reasons, the subjective reasons contributing to sleep difficulties were reported as pain, hypervigilance, hyperarousal, nightmares, and an avoidance of sleep due to a fear of nightmares [42].

It was also demonstrated that pain served as a negative factor for sleep quality and anxiety symptoms in this study. Body pain was found to reduce sleep quality, result in negative coping strategies, increase anxiety symptoms, and eventually decrease HRQoL; in turn, poor sleep quality could also enhance the experience of pain or contribute to a lower pain threshold [43]. However, the problems of bodily pain and its negative effects were underestimated and ignored in the Chinese military. Injuries and pain occurred frequently when high-intensity physical training was required, though inappropriate training methods or procedures were not used. However, the number of those who approached health professionals was only a small portion of those who truly suffered from chronic pain. A majority of servicemen chose to suffer pain in silence for fear of the stigma associated with reporting pain. In Chinese military culture, a soldier is often regarded as symbol of power, strength and reliability; complaining about pain would involve being stigmatized as being weak, incapable or less fit for promotion [44]. As a result, instead of complaining about pain, soldiers may address their sleep complaints because sleep problems are regarded as medical problems in nature. Therefore, the evaluation of pain is important and encouraged when treating soldiers who complain about sleep difficulties. Additionally, adding pain management information into regular health education for the military could be a normalizing intervention to reduce the discrimination related to medical visits due to pain.

This study contributed to the current evidence that negative coping strategies and poor sleep quality could result in anxiety symptoms, with the supporting results including the positive correlation between the NC and SAS scores, between the NC and PSQI and between the PSQI and the SAS (Table 4) and the results of the regression analysis (Table 5). The participants with insufficient sleep were more likely to adopt negative coping strategies such as “restraint or suppression” or “denial”, which further aggravated anxious symptoms.

Several limitations of this study should be noted. First, the sample size was not large enough to include more female soldiers and more military personnel from different ranks. This may reduce the positive outcomes when examining the impacts of demographic variables on sleep quality and HRQoL. Another limitation was the data being collected during a relative peacetime, which limits the results’ application to military personnel on deployment or in combat. Moreover, the ability to examine the relationships between cause and effect for all factors was limited due to the cross-sectional study design.

## Conclusions

This study examined the current status of sleep quality, anxiety and HRQoL in a Chinese military population from an austere environment. In summary, sleep disorders were a prevalent but underestimated problem in the military, which negatively influenced the overall HRQoL, especially in terms of physical and social functioning. The evaluation of pain and education on pain management were recommended because body pain and its negative impacts on sleep quality, coping strategies, anxious emotions and HRQoL have been found.

## Data Availability

The data used and/or analyzed during the current study are available from the corresponding author on reasonable request.

## Abbreviations

PTSD: Post-traumatic stress disorder
HRQoL: Health-related quality of life
PSQI: Pittsburgh Sleep Quality Index
SAS: Self-rating Anxiety Scale
MOS-SF-36: Medical Outcomes Study Short Form-36
SQ: Subjective sleep quality
SB: Sleep disturbances
SM: The use of sleep medication
SE: Habitual sleep efficiency
SL: Sleep latency
SD: Sleep duration
DD: Daytime dysfunction due to disrupted sleep
TCSQ: Trait Coping Style Questionnaire
PCS: Physical Component Summary
MCS: Mental Component Summary
GH: General health
RP: Role limitations due to physical health (role-physical)
BP: Bodily pain
PF: Physical functioning
MH: Mental health
VT: Vitality
SF: Social functioning
RE: Role limitations due to emotional problems
NC: Negative coping
PC: Positive coping
